# Genomic Prediction Using Alternative Strategies of Weighted Single-Step Genomic BLUP for Yearling Weight and Carcass Traits in Hanwoo Beef Cattle

**DOI:** 10.3390/genes12020266

**Published:** 2021-02-12

**Authors:** Hossein Mehrban, Masoumeh Naserkheil, Deuk Hwan Lee, Chungil Cho, Taejeong Choi, Mina Park, Noelia Ibáñez-Escriche

**Affiliations:** 1 Department of Animal Science, Shahrekord University, Shahrekord 88186-34141, Iran; hosseinmehrban@gmail.com; 2 Department of Animal Science, University College of Agriculture and Natural Resources, University of Tehran, Karaj 77871-31587, Iran; naserkheil@ut.ac.ir; 3 Department of Animal Life and Environment Sciences, Hankyong National University, Jungang-ro 327, Anseong-si 17579, Gyeonggi-do, Korea; 4 Hanwoo Genetic Improvement Center, NongHyup Agribusiness Group Inc., Seosan 31948, Korea; blup82@gmail.com; 5 Animal Breeding and Genetics Division, National Institute of Animal Science, Rural Development Administration, Cheonan 31000, Korea; Choichoi6695@korea.kr (T.C.); mina0412@korea.kr (M.P.); 6 Institute for Animal Science and Technology, Universitat Politècnica de València, 46022 València, Spain; noeibes@dca.upv.es

**Keywords:** carcass traits, yearling weight, weighted single-step genomic procedures, SNP window, Hanwoo cattle

## Abstract

The weighted single-step genomic best linear unbiased prediction (GBLUP) method has been proposed to exploit information from genotyped and non-genotyped relatives, allowing the use of weights for single-nucleotide polymorphism in the construction of the genomic relationship matrix. The purpose of this study was to investigate the accuracy of genetic prediction using the following single-trait best linear unbiased prediction methods in Hanwoo beef cattle: pedigree-based (PBLUP), un-weighted (ssGBLUP), and weighted (WssGBLUP) single-step genomic methods. We also assessed the impact of alternative single and window weighting methods according to their effects on the traits of interest. The data was comprised of 15,796 phenotypic records for yearling weight (YW) and 5622 records for carcass traits (backfat thickness: BFT, carcass weight: CW, eye muscle area: EMA, and marbling score: MS). Also, the genotypic data included 6616 animals for YW and 5134 for carcass traits on the 43,950 single-nucleotide polymorphisms. The ssGBLUP showed significant improvement in genomic prediction accuracy for carcass traits (71%) and yearling weight (99%) compared to the pedigree-based method. The window weighting procedures performed better than single SNP weighting for CW (11%), EMA (11%), MS (3%), and YW (6%), whereas no gain in accuracy was observed for BFT. Besides, the improvement in accuracy between window WssGBLUP and the un-weighted method was low for BFT and MS, while for CW, EMA, and YW resulted in a gain of 22%, 15%, and 20%, respectively, which indicates the presence of relevant quantitative trait loci for these traits. These findings indicate that WssGBLUP is an appropriate method for traits with a large quantitative trait loci effect.

## 1. Introduction

In the last decade, the two-step genomic best linear unbiased prediction (GBLUP) method [1] has been the statistical method routinely used for genomic evaluations due to its low computational demand. This method assumes that single-nucleotide polymorphisms (SNPs) effects are normally distributed with equal variance [2,3] and for many polygenic traits, performs as well as Bayesian approaches [4,5,6,7], such as BayesA, BayesB, and BayesL, assuming heterogeneous variances of SNPs effects [2,8]. However, for traits controlled by few quantitative trait loci (QTL) with large effects and many QTL with small or null effects, the GBLUP underperforms. Therefore, a termed weighted GBLUP (WGBLUP) with locus-specific variance, including different weights for SNPs, in the construction of the genomic relationship matrix **G** was proposed [9]. Many studies identified that WGBLUP outperformed GBLUP [10,11,12,13]. WGBLUP and Bayesian procedures were also not considerably different in terms of accuracy for specific traits influenced by few QTL or traits known for being influenced by important QTL [9,13,14]. In both GBLUP and WGBLUP methods, the phenotypic information from non-genotyped animals cannot be utilized, resulting in frequently reduced accuracy and increased bias of these methods [15,16,17,18]. Moreover, in practice, all individuals cannot be genotyped; therefore, the single-step GBLUP (ssGBLUP) methodology was developed to overcome those challenges [19,20,21]. In the past few years, an alternative approach based on the WGBLUP and ssGBLUP framework was proposed by Wang et al. [22], termed the weighted single-step genomic BLUP (WssGBLUP). Zhang et al. [6] developed several iterative weighting strategies with simulation data and indicated that the use of a common weight as a window could improve the accuracy of prediction compared with single SNP weighting. Also, Teissier et al. [23] and Oget et al. [24] depicted that WssGBLUP and its alternatives performed more accurately than ssGBLUP in French dairy goats and sheep. 

In Hanwoo beef cattle, carcass traits (backfat thickness (BFT), carcass weight (CW), eye muscle area (EMA), and marbling score (MS)), and yearling weight (YW) are economically important traits used to select young and proven bulls [25]. Since there is enriched traditional pedigree information available in Hanwoo [26], it is expected that the use of distinct WssGBLUP alternatives [6] can be utilized to improve genomic prediction accuracy for traits with different genetic architectures [7]. Nonetheless, these methods have not yet been comprehensively investigated in breeding schemes for this breed. Therefore, this study aimed to investigate the effect of SNP weighting on the accuracy of genomic evaluation and compare it with those obtained with the unweighted ssGBLUP and pedigree-based BLUP methods for studied traits in Hanwoo beef cattle.

## 2. Materials and Methods

### 2.1. Ethics Statement

Genomic data, pedigree and phenotypic data related to growth and carcass traits, were generated following the protocol for the progeny test program, as notified by the Ministry of Agriculture, Food and Rural Affairs based on livestock law in Korea. Hanwoo Improvement Center (HIC) of the National Agricultural Cooperative Federation, as an enforcement institution for the testing program for selecting proven Hanwoo bulls, is obligated to maintain data and ownership of enrolled animals under notice. DNA samples were obtained from blood samples collected by veterinarians. Pedigree data were recorded by the Korean Animal Improvement Association, data for growth traits were obtained from the Hanwoo Improvement Center (http://www.limc.co.kr (accessed on 11 February 2021)), and data for carcass traits were recorded by special inspectors, from the Institute of Korean Animal Products Evaluation (http://www.ekape.or.kr (accessed on 11 February 2021)), at the slaughterhouse through the progeny testing program in Korea.

### 2.2. Phenotypic and Pedigree Data

The records of YW for 15,796 animals (10,114 bulls and 5682 steers) and carcass traits of 5622 steers were born between 1997 and 2017 and raised in Hanwoo Improvement Center of the National Agricultural Cooperative Federation were used for this study (Table 1). The pedigree data of 54,284 animals, obtained after tracing the pedigree file back 11 generations, utilized in the animal model. MS was measured using a categorical system of nine classes ranging from the lowest score of one (no marbling) to the highest score of nine (abundant marbling). The carcass traits were measured according to the Korean carcass grading system in steers at approximately 24 months of age, ribbed between the thirteenth rib and the first lumbar vertebrae, and 24 h postmortem, according to notification No. 2014-4 of the Ministry of Agriculture, Food and Rural Affairs. YW for each animal was determined from the weight (W_t_) at the termination (t) of the test (body weight at ~12 months) and the previous weight (W_t − 1_) recorded at a time point (t_−1_) before (body weight at ~6 months) the termination, according to the equation described by Park et al. [27]:

$$\mathrm{YW}=\;\left[\left(\frac{W_{t}\;-{\;W}_{t_{-1}}}{t\;-{\;t}_{-1}}\right)\;\times \;\left(365-{\;t}_{-1}\right)\right]+{\;W}_{t_{-1}}\;$$

### 2.3. Genotypic Data

The dataset consisted of 12,764 animals (call rate > 90%) genotyped using Illumina BovineSNP50K version 2 (*n* = 3720), version 3 (*n* = 4121) and customized Hanwoo version 1 (*n* = 4923). The genotyped animals with Illumina BovineSNP50K version 2 were considered as reference populations to impute target animals (The genotyped animals using Illumina BovineSNP50K version 3 and customized Hanwoo version 1) with FImpute V3 [28]. There were 52,791 SNPs total after imputation on the 29 chromosomes. The analyses included genotypes for steers with phenotypes and their sires. Genotypic data were available for 5134 and 6616 animals for carcass traits and YW, respectively. SNPs with minor allele frequencies lower than 0.01 (8818 SNPs) and a maximum difference between the observed and expected frequency of 0.15 as a departure of heterozygous from the Hardy-Weinberg equilibrium (23 SNPs) were removed, resulting in 43,950 SNPs. 

### 2.4. Statistical Analyses

#### 2.4.1. Adjusted Phenotypes

The phenotypes were adjusted for fixed effects based on the classical multi-trait animal model (with pedigree and whole phenotype data) as proposed by Lee et al. [18] through AIREMLF90 software [29] as follows

(1) y=Xb+Zu+e

where **y** is the vector of observations for the trait of interest; **b** is the vector of fixed effects, including batch-sex (87 levels) and birth place (109 levels) for YW; slaughter date (274 levels), and slaughter age (days from birth to slaughter) was considered as covariates for carcass traits; **u** is the vector of random genetic additive effects; **e** is the vector of random residual effects; **X** and **Z** are incidence matrices related to fixed and random genetic additive effects, respectively. Var (**u**) = **G**⊗**A** and Var(**e**) = **R**⊗**I** were assumed, where **A** is the numerator relationship matrix, **I** is the identity matrix, and **G** and **R** are additive genetic and residual covariance, respectively, for the five traits (Tables S1–S3).

Therefore, the adjusted phenotype for each animal was calculated as the sum of the estimated breeding values (EBVs), and the residual [18,30].

#### 2.4.2. Traditional Evaluation

The single-trait pedigree-based evaluations (PBLUP) were performed using the following animal model

(2) $$y_{\mathrm{adj}}=1\mu +Za+e$$

where $y_{\mathrm{adj}}$ is the vector of observations for the trait adjusted for fixed effects according to the equation (1); **1** is a vector of ones; µ is the overall mean; **a** is the vector of random genetic additive effects; **e** is the vector of random residual effects; **Z** is incidence matrix related to random genetic additive effects. Var (**a**) = **A**$\sigma_{a}^{2}$ and Var (**e**) = **I**$\sigma_{e}^{2}$ were assumed, where **A** is the numerator relationship matrix, **I** is the identity matrix, and $\sigma_{a}^{2}$ and $\sigma_{e}^{2}$ are additive genetic and residual variance, respectively, for each trait.

#### 2.4.3. Genomic Evaluation 

In the ssGBLUP method, the statistical model was the same as that for the traditional evaluation; however, the non-genotyped and the genotyped animals were simultaneously included in the hybrid relationship matrix of **H** as a combination of **A** (numerator relationship matrix) and **G** matrices. The inverse of the **H** matrix was obtained as the following equation [20,31] employing preGSf90 software [32]

(3) $$H^{-1}=A^{-1}+\;\left[\begin{array}{cc} 0 & 0\\ 0 & {\left(0.095G+0.05A_{22}\right)}^{-1}-A_{22}^{-1} \end{array}\right]$$

where **A**_22_ is the numerator relationship matrix for genotyped animals. 

The genomic relationship matrix (**G**) was constructed as described by VanRaden [3]

(4) $$G=\;\frac{MDM^{\prime }}{2\sum_{i=1}^{m}p_{i}\;\left(1-p_{i}\right)},$$

where m is the total number of markers (43,950), p_i_ is the allelic frequency of ith marker, **M** is the matrix of centered genotypes and **D** is the identity matrix. 

For the WssGBLUP method, **D** is a diagonal matrix of weights for markers which was obtained with back-solved markers effect (**û**) from genomic estimated breeding values (GEBVs) (**â**) as follows [22]:

(5) $$\hat{u}=\lambda DM^{\prime }G^{-1}\hat{a}.\;\lambda =\;\frac{1}{2\sum_{i=1}^{m}p_{i}\;\left(1-p_{i}\right)}$$

In this stage, GEBV was replaced by direct genomic values (DGV), which was obtained as follows [20]

(6) $${\mathrm{DGV}}_{i}=-\left(\underset{j\neq i}{\sum }g^{\mathrm{ij}}{\;\mathrm{GEBV}}_{j}/g^{\mathrm{ii}}\right)$$

where g^ij^ is the elements in **G**^−1^ corresponding to relationships between animal i and j. It must be noted that DGV is a more relevant starting point than GEBV for calculating SNP effects because genotyped populations may include animals with different levels of accuracy [33].

In the next step, each diagonal element of **D** was replaced through the normalization of weighting strategies [6] to highlight regions of higher impact on the genetic variation of the studied traits. Then, the **G** matrix was created based on the new **D** matrix and combined with **A** to constitute a new **H** matrix used to estimate GEBVs and DGVs in a single-step procedure. Updating the DGV and marker effects were continued for 10 iterations in each trait. As proposed by Zhang et al. [6], weighting strategies were considered to calculate the weight for SNPs in the diagonal of **D** matrix (d_ii_) as follows:

(1) Default: SNP weights are calculated based on the individual genetic variance of SNP (d_ii =_ ${\hat{\sigma }}_{u_{i}}^{2}=2p_{i}(1-p_{i})\;{\hat{u}}_{i}^{2}$).

(2) Constant: the maximum genetic variance of SNP effect in the first iteration (c) was estimated. Then the fixed value of c was added to the ${\hat{\sigma }}_{u_{i}}^{2}$ (i.e., ${\hat{\sigma }}_{u_{i}}^{2}+c$) in succeeding iterations.

(3) Nonlinear A: the diagonal elements of **D** matrix (d_ii_) for the nonlinear A was defined as

(7) $$d_{\mathrm{ii}}\;=\;{\mathrm{CT}}^{\frac{\left|{\hat{u}}_{i}\right|}{\mathrm{sd}\left(\hat{u}\right)}-2},$$

where CT (1.125 or 1.25) indicates the departure from normality, $\left|{\hat{u}}_{i}\right|$ is the absolute estimated SNP effect for marker i, and $\mathrm{sd}\left(\hat{u}\right)$ is the standard deviation of the vector of estimated SNPs effect [3]. Besides, the maximum change in SNP variance is limited to five and 20 [11,34].

(4) Largest window: using the concept of SNP-window weights, with weights for a group of (n) SNPs as d_ii_ = max(${\hat{\sigma }}_{u_{i}}^{2}$, ${\hat{\sigma }}_{u_{i+\left(n-1\right)}}^{2}$).

(5) Mean window: the weight for a group of SNPs was calculated as d_ii_ =$\sum_{i=1}^{n}\frac{{\hat{\sigma }}_{u_{i}}^{2}}{n}$.

(6) Summed window: the summation of the individual genetic variance of markers effect for a group of SNPs was calculated as d_ii_$=\sum_{i=1}^{n}{\hat{\sigma }}_{u_{i}}^{2}$.

To optimize window size, four groups of SNPs (25, 50, 75, and 100) were considered for the largest, mean, and summed window weighting strategies. The explained genetic variance for the group of SNPs (k) was obtained as $\frac{var(\sum_{i=1}^{k}Z_{i}{\hat{u}}_{i})}{\sigma_{a}^{2}}\times 100$ [5].

Breeding values, direct genomic values (DGV), and markers effect were estimated utilizing the BLUPF90 programs [29]. A single-step genome-wide association study (ssGWAS) was conducted using model (1) with both ssGBLUP and WssGBLUP methods to reveal the architecture of the studied traits. 

The variance components for traditional and genomic prediction were estimated using the pedigree-based single-trait animal model (Table S4); because the univariate model was performed to obtain EBV/GEBV.

### 2.5. Accuracy of the Genetic Evaluations

Accuracies of all the methods were evaluated by splitting the whole data into training and validation dataset by a cut-off point of birth year. The training set included the pedigree, the adjusted phenotype (obtained from Equation (1)) of non-genotyped animals, and the genotype of 4960 animals for YW (4331 for carcass trait) born between 1997 and 2015. The validation set contained 803 and 1656 animals for carcass traits and YW, respectively born between 2016 and 2017 in which the adjusted phenotypes were assumed to be unknown, and only the genotypes and pedigree information was retained. The predicted ability of different methods was calculated as the correlation between adjusted phenotypes and EBV/GEBV of animals in the validation populations. Accuracy was determined as the predicted ability divided by the root of heritability, which was achieved through the single-trait pedigree-based animal model. The bias was defined as a regression coefficient of the adjusted phenotypes on EBV/GEBV. The root of mean square error (RMSE) was predicted as the mean of the squared differences between adjusted phenotype and EBV/GEBV.

## 3. Results 

### 3.1. Comparisons of Alternative WssGBLUP Approaches Over Iterations

#### 3.1.1. Single Weighting Procedures

**Figure 1:**
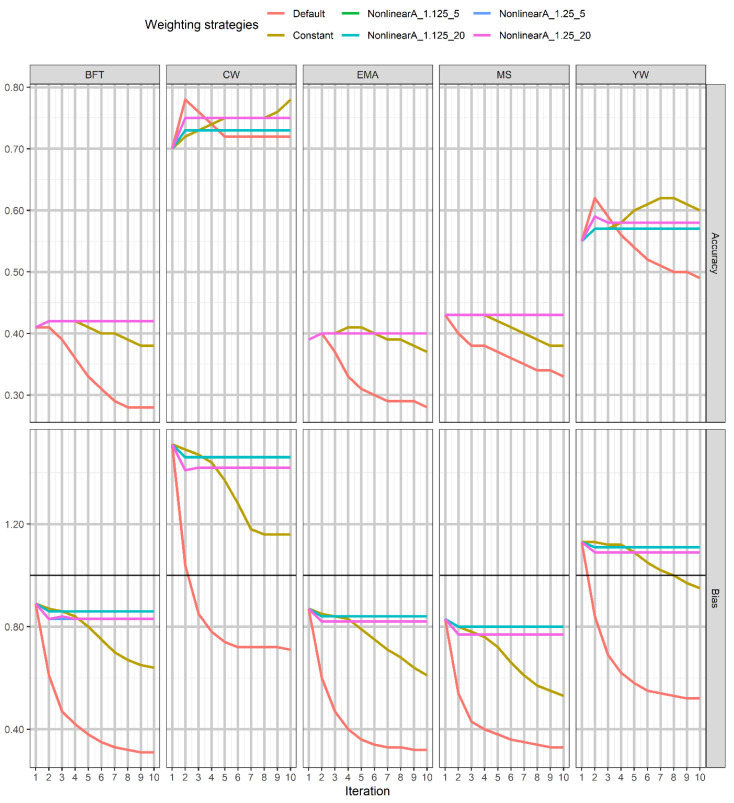
The trend in prediction accuracy and bias of genomic estimated breeding value (GEBV) was obtained using single weighting strategies across iterations for the studied traits. The CT (departure from normality) and exponent limits are shown as NonlinearA_CT_limit in Nonlinear A methods. BFT, backfat thickness; CW, carcass weight; EMA, eye muscle area; MS, marbling score; YW, yearling weight.

The accuracies of genomic prediction for YW and carcass traits using linear (default and constant) and different nonlinear A weighting approaches over ten iterations are presented in Figure 1. The first iteration corresponded with ssGBLUP where the SNPs weights were equal to one. The highest accuracies using default weighting were obtained at the second iteration for EMA, YW, and especially for CW traits and then deteriorated over the succeeding iterations, while for BFT and MS, the accuracy decreased after iteration 1. The results showed that the greatest accuracy using the constant method achieved for EMA and YW at iteration 5 and 7, respectively, and the trend toward increased for CW was observed over iterations. However, the accuracy for BFT and MS remained stable between iterations 1 and 4 and then declined over the later iterations. Similar accuracies were generally derived from the nonlinear A methods with CT values of 1.125 and 1.25 and exponential limit of 5 and 20 for BFT, EMA, and MS over iterations; although, we observed a slight increase in accuracy for BFT and EMA at the second iteration. The results showed that the nonlinear A methods with different CT values resulted in different magnitudes of accuracy for CW and YW, regardless of considering limiting the maximum SNPs variances. Notably, the highest accuracy using nonlinear A with a CT value of 1.25 was obtained at the second iteration for CW and YW. According to the results, the maximum accuracy was observed using the default method for CW and YW at the second iteration and with the constant method for MS at iteration 2 and EMA at iteration 5 as well as using nonlinear A with the CT value of 1.25 and limiting the maximum SNP variance of 5 (nonlinearA_1.25_5) at iteration 2 for BFT trait.

The biases of genomic prediction for the traits under study using different single SNP weighting methods over ten iterations are shown in Figure 1. Based on our results, the rapid incline of bias using the default method over iterations was observed for the studied traits, whereas in the constant method, no sudden drop in the bias was noted over ten iterations. A stable trend was seen in the nonlinear A methods along with ten iterations in all traits under study. 

In terms of the RMSE ratio, the trend toward increased in all over iterations using the default method was obtained for BFT, EMA, and MS (Figure S1). 

However, we observed an increase or a decrease of the RMSE ratio by this method for CW and YW in which the lowest ratio was at the second iteration. Different trends using the constant method depending on the trait were observed so that the RMSE ratio for BFT, EMA, and MS gradually increased after iteration 5, 7, and 4, respectively. Nonetheless, the lowest RMSE ratio was obtained at iteration 6 for CW and iteration 4 for YW and then remained constant over the succeeding iterations. The results also showed that the RMSE ratio using the nonlinear A methods remained constant over ten iterations for all traits, except for CW and YW in which a slight drop between iterations 1 and 2 was observed.

#### 3.1.2. Window Weighting Procedures 

**Figure 2:**
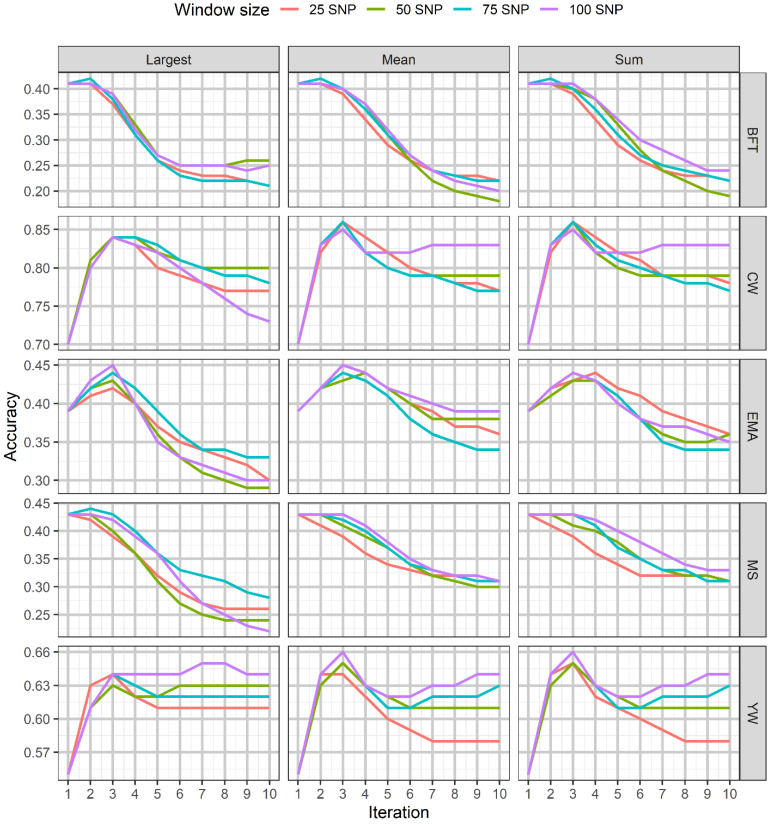
The trend in prediction accuracy of GEBV obtained using window weighting strategies with varying window sizes across iterations for the studied traits. BFT, backfat thickness; CW, carcass weight; EMA, eye muscle area; MS, marbling score; YW, yearling weight.

**Figure 3:**
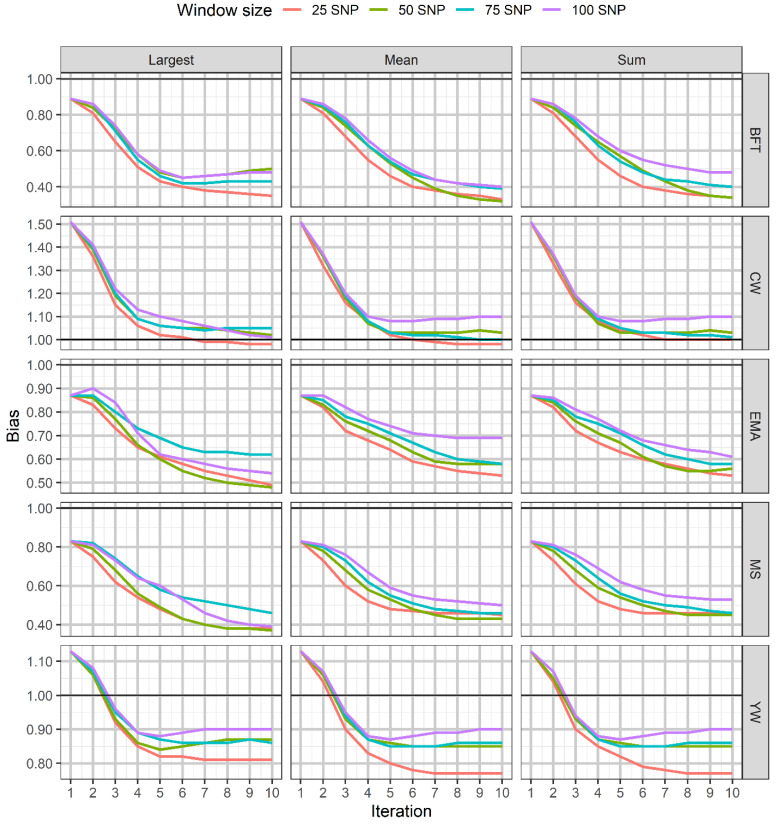
The trend in the bias of GEBV obtained using window weighting strategies with varying window sizes across iterations for the studied traits. BFT, backfat thickness; CW, carcass weight; EMA, eye muscle area; MS, marbling score; YW, yearling weight.

The accuracies obtained using SNPs window weighting methods (largest, mean, and summed) with different window sizes over ten iterations for all traits are illustrated in Figure 2. The results of the largest window procedure with a window size of 75 SNPs showed a slightly enhanced accuracy of prediction for BFT and MS at iteration 2, while no improvement for MS using mean and summed window methods was noted over the iterations. For the other traits, the highest accuracy was obtained at iteration 3 and then declined across the succeeding iterations in the methods under study. The results also indicated that the highest accuracies using the window procedures occurred for EMA using the largest window (window size of 100 SNPs) and with summed window weighting (windows size of 75) for both CW and YW traits. Moreover, window weighting methods tended to give upward bias across iterations for all traits except for CW (Figure 3). The trends of RMSE ratio with the various methods and windows size were different over iterations in the five traits.

For BFT and MS, increasing trends in this ratio were observed along with iterations, while for other traits, the lowest magnitudes of ratio occurred at iterations 2, 3, and 4 depending on windows size and weighting strategies (Figure S2).

### 3.2. Comparisons of Pedigree-Based BLUP, ssGBLUP, and WssGBLUP

**Figure 4:**
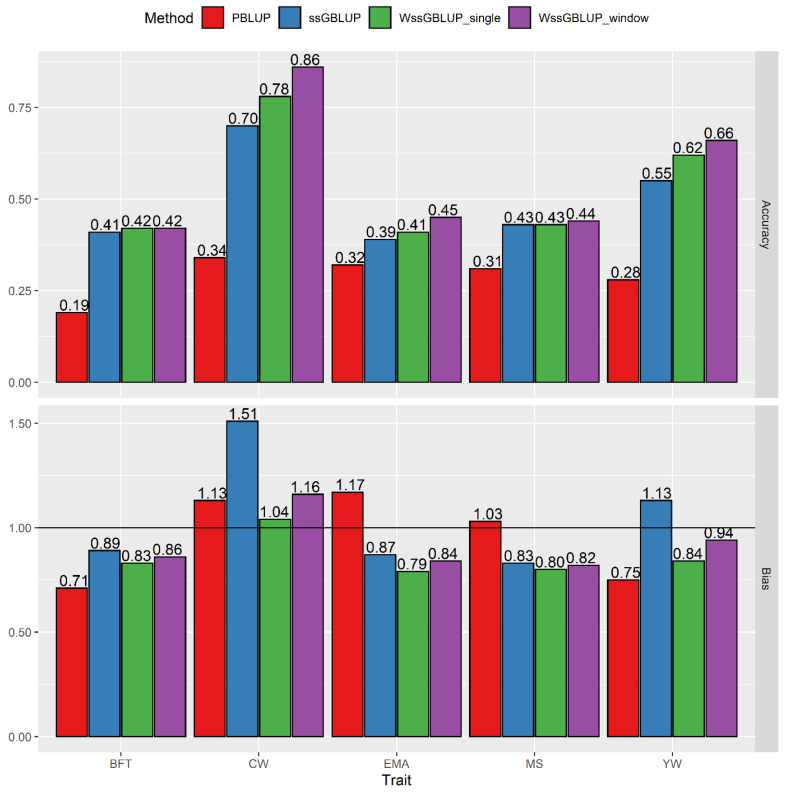
Prediction accuracy and bias of GEBV/estimated breeding value (EBV) obtained using pedigree-based best linear unbiased prediction (PBLUP), single-step genomic best linear unbiased prediction (ssGBLUP), and the best single and window weighting ssGBLUP (WssGBLUP) for the studied traits. BFT, backfat thickness; CW, carcass weight; EMA, eye muscle area; MS, marbling score; YW, yearling weight. The default method for CW and YW at the second iteration, the constant method for MS and EMA at iteration 2 and 5, respectively, and nonlinear A with CT value of 1.25 and limiting the maximum SNP variance of 5 (nonlinearA_1.25_5) at iteration 2 for BFT were the best single weighting WssGBLUP method. The summed window (window size = 75) for CW and YW at iteration 3, The largest window (window size = 75) for BFT and MS at iteration 2, and the largest window (window size = 100) at the third iteration for EMA were the best window weighting WssGBLUP method.

The accuracies of genomic prediction for the five traits obtained with pedigree-based BLUP, ssGBLUP, and the best weighting procedure of WssGBLUP are shown in Figure 4. The results showed that the ssGBLUP method substantially outperformed PBLUP models with the same phenotypic and pedigree data for carcass traits and yearling weight. Accuracies with ssGBLUP (PBLUP) were 0.41 (0.19), 0.70 (0.34), 0.39 (0.32), 0.43 (0.31), and 0.55 (0.28) for BFT, CW, EMA, MS, and YW, respectively (Figure 4). The accuracies derived from the WssGBLUP using both single and window weighting methods were more accurate than ssGBLUP for CW, EMA, and YW, whereas a slight improvement for BFT and MS was observed. In other words, compared to ssGBLUP, the gain was 23% (11%) for CW, 15% (5%) for EMA, and 20% (13%) for YW using the window weighting (single weighting) method. Overall, the window weighting performed better than single weighting for CW (11%), EMA (11%), MS (3%), and YW (6%), while no gain was observed for BT.

The regressions of adjusted phenotype on the estimated breeding value ranged from 0.71 to 1.17 for PBLUP, 0.83 to 1.51 for ssGBLUP, 0.79 to 1.04 for WssGBLUP_single, and 0.82 to 1.16 for WssGBLUP_window methods (Figure 4). Predictions from genomic methods were indicated to have less bias than predictions from PBLUP for BFT, EMA, and YW. Besides, the WssGBLUP methods improved unbiasedness for CW and YW than the ssGBLUP model; however, differences between the regression coefficients were trivial for BFT, EMA, and MS.

Except for EMA, RMSEs were decreased by switching PBLUP to ssGBLUP or WssGBLUP (Table S5). The improvement in accuracy for CW, EMA, and YW in the window strategy of WssGBLUP compared with those obtained from ssGBLUP was investigated through a single-step GWAS. The results showed that the proportions of genetic variance explained by the window (1 Mb) were trivial in the ssGBLUP for all traits. However, the WssGBLUP (window strategy) detected genome-wide highly significant SNPs, particularly on chromosomes 6 and 14 for CW, EMA, and YW (Figure S3).

## 4. Discussion 

This study aimed to assess the accuracy of genomic predictions for yearling weight and carcass traits using WssGBLUP alternatives in Hanwoo beef cattle. A previous study was performed for genomic evaluation using WssGBLUP with only default weighting in this breed [18]. Recently, Lopez et al. [11] and Heras-Saldana et al. [12] investigated the effect of single SNP weighting GBLUP (nonlinear A and default procedures) on the accuracy of genomic predictions without including the pedigree in the analyses of the same breed. These studies neither considered the genomic evaluation of YW nor comprehensively examined various weighting strategies on the accuracy of genomic breeding values. Presently, YW is one of the economically important traits involving in the appreciation and profitability of the meat used for selecting young bulls in the Hanwoo breeding scheme [25]. 

According to the observed results (Figure 4), the highest accuracy was obtained at the third iteration for CW, EMA, and YW, whereas for BFT and MS achieved at the second iteration using window WssGBLUP. The results of most studies showed that maximum accuracy is achieved at the second iteration. For instance, Wang et al. [22], Teissier et al. [23,35], and Oget et al. [24] exhibited that the most accurate genomic evaluation was obtained at the second iteration. Nonetheless, the highest accuracy of genomic prediction was observed at the third [12] and the fourth [11,12] iteration for BFT and CW in Hanwoo beef cattle. It is highlighted that the increase in accuracy can occur in higher iteration when traits are controlled by a small number of genes or affected by major genes. In a study using simulation, Zhang et al. [6] reported that the number of iteration affecting the maximum accuracy depends on the number of QTL. They indicated that improvement in the prediction accuracy for 5- and 100-QTL scenarios were observed at iterations 3 and 4, respectively, while no gain in accuracy with increasing iteration was obtained for the 500-QTL scenario. Similar results were observed by Lourenco et al. [36], who realized the highest accuracy obtained at the third iteration across the different number of genotyped animals when the traits are controlled by 10 or 50 QTL. Recently, Lu et al. [13] reported that the accuracy of genomic prediction using WGBLUP was the highest at the fourth iteration for resistance to *Streptococcus agalactiae* in GIFT strain of *Oreochromis niloticus*.

Overall, the genomic prediction deteriorated with increasing iteration, except for constant and particular nonlinear A weighting, which were more persistent than other weighting procedures. The explanation of the observed decline in accuracy with iteration for most of the methods could be because of the overweighting or underweighting of the SNPs across iterations [22,35]. In other words, the decrease in accuracy of GEBV over iterations was due to continuously adding weights to the SNPs with large influence while shrinking the SNPs with small effects [6,23]. In contrast, the constant method did not show considerable accuracy changes and SNPs shrinkage because of the addition of a fixed value as the greatest SNP variance at the first iteration [6]. Similar to our study, Lopez et al. [11] and Fragomeni et al. [37] achieved decreasing trends in the accuracy of genomic prediction using the default weighting GBLUP. However, the limitation on the maximum change in weight of SNP could result in stable genomic prediction after the second iteration in the nonlinear A methods, which corresponded to Fragomeni et al. [37], Lopez et al. [11], and Heras-Saldana et al. [12].

Figure 4 displays the accuracy of genomic predictions obtained using PBLUP, ssGBLUP, and the best WssGBLUP methods (single and window weighting) for all the considered traits. The results showed that the accuracy of the single-step approaches was higher than those of pedigree-based BLUP for all traits, regardless of using weighted or non-weighted models. It was clear that the inclusion of genomic information in the analysis can significantly improve prediction accuracy relative to traditional pedigree-based models due to capturing variation in Mendelian sampling [16]. The superiority of ssGBLUP over pedigree-based BLUP or GBLUP approaches for the prediction of breeding values has been reported in previous studies on Hanwoo cattle [18,26,38] and other beef cattle breeds [30,33,39]. Other studies on French dairy goats [23,40] and sheep [24] identified that ssGBLUP was more accurate than either the pedigree-based BLUP or GBLUP for milk production traits, udder type traits, and somatic cell scores. 

The degree of superiority of the models used for genomic prediction depends on the genetic architecture of the traits of interest [7,35]. The WssGBLUP methods are more flexible than other methods and could set more weight to SNPs that are associated with QTL with a relatively large effect [6]. Our results showed that WssGBLUP outperformed ssGBLUP in terms of prediction accuracy for EMA, YW, and especially CW. These gains in prediction accuracy were consistent with those achieved by Lee et al. [18], who reported the improved accuracy of 7% for CW and 2% for EMA in Hanwoo beef cattle. Also, Lopez et al. [11] and Heras-Saldana et al. [12] demonstrated that weighted versus unweighted GBLUP could be more accurate for BT (5%; 4%) and CW (3%; 2%) rather than EMA and MS in the same breed. Several factors may be responsible for the discrepancy of prediction accuracy between present findings and those of Lopez et al. [11] and Heras-Saldana et al. [12], such as differences in the data structure, the age of the slaughter, and the number of animals genotyped. Compared to this study, they used approximately two times genotyped data which was collected from the steers slaughtered at 30 months of age in commercial farms, while our carcass data was obtained from steers of candidate bulls and animals, including in the official progeny test program, which slaughtered at 24 months of age.

Our findings were supported by results of ssGWAS which reveals major QTLs on chromosomes 6 and 14 for EMA, YW, and particularly for CW (Figure S3), in which the improvement of the prediction accuracy using the WssGBLUP method was clear. The percentage of additive genetic variance explained on chromosome 14 (6) was 36% (4.56%) for CW, 9.39% (2.60%) for EMA, and 11.64% (9.05%) for YW, which was confirmed by the findings of the previous study [41]. The results of that study showed that some genomic regions on chromosome 14 were to influence CW, EMA, and YW, which indicated the same genes were controlling these traits [41]. 

Previously, Lee et al. [42] identified one major QTL on chromosome 14 that is significantly associated with carcass weight in Hanwoo cattle. Also, some studies were shown that there are a few QTL with a large effect for CW [7,18] and EMA [18] in this breed. Our results are also in concordance with earlier reports on different species, which highlights that for quantitative traits that are controlled by a few genes with a moderate to large effect, WssGBLUP outperforms ssGBLUP. For instance, Lourenco et al. [43] reported that WssGBLUP increased the accuracy of genomic evaluation more efficiently than GBLUP for traits with QTL with large effects on dairy cattle. A similar conclusion was given by Tiezzi and Maltecca [10], who reported that gain in reliability was achieved for traits that are controlled by a small number of QTL (fat and protein percentage) in Holsteins when using the weighted G matrix. Besides, Lourenco et al. [36] showed that WssGBLUP could outperform ssGBLUP for less polygenic traits. Furthermore, Vallejo et al. [44] investigated the efficiency of WssGBLUP for bacterial cold water disease resistance and reported an improvement of accuracy (4 percentage points) relative to ssGBLUP. Likewise, Teissier et al. [23] achieved an up to 3% increase in the accuracy of genomic prediction on protein content using WssGBLUP over ssGBLUP, as the α_S1_-casein gene is well known to segregate in the Alpine and Saanen breeds. In a recent study, Oget et al. [24] also showed that WssGBLUP performed more accurately than ssGBLUP, ranging from 2.06% to 8.75% for production, somatic cell score, and type traits which were affected by the major gene SOCS2 in Lacaune dairy sheep. On the other hand, accuracies from ssGBLUP and WssGBLUP analyses were near the same for BT and MS (Figure 2). This indicates that using weights for SNPs when the quantitative trait has a polygenic inheritance and follows the infinitesimal model would not benefit from the accuracy of genomic evaluation compared with ssGBLUP. In this respect, Teissier et al. [35] concluded that for polygenic traits, the same accuracy could be obtained with the ssGBLUP and WssGBLUP method, which is in complete agreement with the findings of our study. Recently, Lu et al. [45] indicated that WssGBLUP was more accurate (6%) than ssGBLUP for Edwardsiellosis resistance in Japanese flounder, which may be due to the presence of QTL on chromosome 14 with large effects for this trait.

In the current study, the impact of the single weighting and window weighting with different window sizes (25, 50, 75, and 100 non-overlap adjacent SNPs) on the accuracy of genomic prediction was investigated. Our findings showed that the window weighting performed better than single ones for CW, EMA, and YW (Figure 4). Also, the highest accuracy was obtained using 100 SNPs for EMA and 75 SNPs for CW and YW, which might be due to QTL size (Oget et al., 2019). In a study using simulation, Hassani et al. [46] showed the worst prediction accuracy occurred using the single SNP method. Besides, it was shown that the window procedures were more capable than single ones to decrease uncertainty [47] and better capturing the signal from the QTL in that region for traits influenced by few QTL with a relatively large effect [6,46]. Previous reports in simulation studies showed that common variances on the partitioning genome region followed by the selection of 100 SNPs within those selected regions resulted in a higher accuracy over one SNP specific variance [14,48]. Also, Teissier et al. [35] investigated a similar approach (WssGBLUP) to the analysis of milk production traits, udder type traits, and somatic cell scores in the two French dairy goat populations and used a window size of 40 SNPs for all traits. Su et al. [47] compared weighted GBLUP models with a region size of 5, 10, 30, 50, 70, 100, and 150 SNPs using a 54K-SNP chip and reported an improvement of the reliabilities up to 1% point using the mean variance of a 30-SNP window for four milk production traits and mastitis in Nordic Holsteins. Similarly, Zhang et al. [6] mentioned that a common weight for a group of 20 adjacent SNPs (over 5, 10, 50, and 100) produced the highest accuracy when the trait of interest is not polygenic. Teissier et al. [23] showed that the optimal length of the window was 40 SNPs among regions size of 2, 5, 10, 20, 40, 80, 100, 150, 200, and 250 consecutive SNPs when largest and sum WssGBLUP was applied for protein content in French dairy goats. In this respect, Oget et al. [24] obtained the best accuracies using an alternative WssGBLUP strategy for each trait individually. They showed that depending on the trait, the best WssGBLUP method was with large window size (100–200 SNPs) for milk, fat, and protein yields, and for somatic cell score, a medium window size (40–45 SNPs) for fat and protein contents, and small window size (1–30 SNPs) for the udder-type traits. In a recent study, Liu et al. [49] reported that using a 54K-SNP set, weighted ssGBLUP model with a common weight on the SNPs within a specific region (30 SNPs) outperformed the ssGBLUP model for milk and protein yields but not for fat yield and three female fertility traits in Danish Jersey cattle.

Consequently, the proper choice of weighting **G** matrix in models seems to be a reasonable approach to attain the greatest accuracy, especially in the presence of QTL with large effects.

## 5. Conclusions

This study aimed to compare pedigree-based BLUP and different genomic evaluation methods for yearling weight and carcass traits. Our results demonstrate that the accuracy of GEBV/EBV estimated with WssGBLUP and its alternatives are more than or as accurate as those from ssGBLUP, followed by PBLUP. The window weighting procedures performed better than single SNP weighting for CW, EMA, MS, and YW, whereas no gain in accuracy was observed for BFT. Also, considering window methods for estimation SNPs weights in WssGBLUP improved the accuracy of genomic predictions for EMA, YW, and particular CW over ssGBLUP, whereas the gain was trivial for BT and MS.

## Figures and Tables

**Table 1:** Summary statistics for phenotypic data used to estimate variance components in Hanwoo cattle.

Trait (Units)	Sample Size	Mean (SE)	Min.	Max.	SD	CV %
Backfat thickness (mm)	5622	9.92 (0.05)	1.00	35.00	3.95	39.83
Carcass weight (kg)	5619	370.48 (0.57)	213.00	562.00	42.80	11.55
Eye muscle area (cm^2^)	5617	81.62 (0.12)	50.00	121.00	8.98	11.00
Marbling score (score)	5622	3.53 (0.02)	1.00	9.00	1.64	46.50
Yearling weight (kg)	15,796	357.13 (0.35)	190.49	547.65	44.07	12.34

SE: standard error; SD: standard deviation; CV: coefficient of variation.

## Data Availability

The data presented in this study are available in [(http://www.limc.co.kr (accessed on 11 February 2021)) and (http://www.ekape.or.kr (accessed on 11 February 2021))].

## Supplementary Materials

The following are available online at https://www.mdpi.com/2073-4425/12/2/266/s1, Table S1: Estimates of genetic variances (diagonal) and covariances (off diagonal) between traits in Hanwoo cattle; Table S2: Estimates of environmental variances (diagonal) and covariances (off diagonal) between traits in Hanwoo cattle; Table S3: Estimates of genetic (upper diagonal) and environmental (lower diagonal) correlations between traits in Hanwoo cattle.; Table S4: Univariate variance components and heritability estimated from pedigree and phenotypic information in Hanwoo cattle; Table S5: The root of mean square error (RMSE) of EBV/GEBV for pedigree-based BLUP (PBLUP), single-step GBLUP (ssGBLUP), single weighting weighted ssGBLUP (WssGBLUP_single) and window weighting WssGBLUP (WssGBLUP_window); Figure S1: Trend the root of mean square error (RMSE) to RMSE ssGBLUP ratio obtained using single weighting strategies across iterations for the studied traits. BFT, backfat thickness; CW, carcass weight; EMA, eye muscle area; MS, marbling score; YW, yearling weight; Figure S2: Trend the root of mean square error (RMSE) to RMSE ssGBLUP ratio obtained using window weighting strategies with varying window size across iterations for the studied traits; Figure S3: Manhattan plots of the proportion of genetic variance (%) explained by 1Mb region for the studied traits using single-step GBLUP and window weighted ssGBLUP approach. BFT, backfat thickness; CW, carcass weight; EMA, eye muscle area; MS, marbling score; YW, yearling weight. The summed window (window size = 75) for CW and YW at iteration 3, the largest window (window size = 75) for BFT and MS at iteration 2, and the largest window (window size = 100) at the third iteration for EMA were the best window weighting WssGBLUP method.
